# Updated reference values in pelvic ultrasonography for a Spanish population of healthy girls between 6 and 12 years old

**DOI:** 10.1002/edm2.233

**Published:** 2021-02-04

**Authors:** Marisa Villalobos Gálvez, Viviana Patricia Beltrán Salazar, Mireia Pérez Adell, Carmina Durán Feliubadalo, Raquel Corripio

**Affiliations:** ^1^ Pediatric Endocrine Department Parc Tauli Sabadell Hospital Universitari Sabadell Spain; ^2^ Pediatric Radiology Department Parc Tauli Sabadell Hospital Universitari Sabadell Spain

**Keywords:** girls, ovarian, pelvic, reference, Spanish, ultrasonography, uterine

## Abstract

**Background:**

Pelvic ultrasonography (PUS) of the uterus and ovaries allows the diagnosis of changes in sexual development. However, the reference values used in Spain originate from old studies conducted in other countries.

**Objective:**

To determine reference uterine and ovarian measurements by PUS and according to pubertal status and bone age in a Spanish population of healthy girls aged between 6 and 12 years.

**Materials and methods:**

Descriptive cross‐sectional study performed on 221 healthy girls from 2017 to 2019. Ovarian and uterine measurements were described and associated with chronological age, bone age, and Tanner stage. ROC curves were used to assess the predictive value of tests for Tanner stage II.

**Results:**

We described reference values for all PUS uterine and ovarian measurements assessed. Subjects in Tanner II (thelarche) had a mean age of 9.7 years (*SD* = 1.1) and mean BMI of 19.2 kg/m^2^. Fundal–cervical ratio changed from 1:1 to 2:1 at 12 years of chronological age (62.5% with 1:1 ratio; *p* < 0.0001) and 13 years of bone age (2:1 in 85.71%, *p* < 0.0001). Mean uterine length for Tanner II was 4.065 cm (*SD* = 0.736); mean ovarian volume was 2.466 cm^3^ (*SD* = 1.719). Bone age, ovarian volume, and uterine length were good predictors of Tanner stage II.

**Conclusion:**

This is the first study providing reference uterine and ovarian PUS values in a Spanish population of healthy girls aged 6 to 12 years. Use of updated data characteristic of a specific population increases the diagnostic accuracy.

## Graphical abstract

The study provides updated pelvic ultrasound reference values for uterine and ovarian measurements. According to our findings, a significant change in uterine length (*p*  =  0.0020), ovarian transverse diameter (*p*  < 0.0001), ovarian anteroposterior diameter (*p*  =  0.0017), and ovarian volume (*p*  =  0.0014) takes place at 10 years of age in girls with normal development, whereas significant changes in anteroposterior diameter of the fundus (*p*  < 0.0001) and the cervix (*p* < 0.0001) occur at 11 years old (chronological age). Our results showed that bone age and ovarian volume in particular are good predictors of Tanner stage 2.

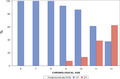

## 1 INTRODUCTION

Pelvic ultrasonography (PUS) is the technique of choice for the evaluation of pediatric genital organs, as an accurate, painless, non‐invasive method that does not require sedation or ionizing radiation.^1^ This method allows the assessment of sexual development, in particular alterations such as premature or delayed puberty, or sexual ambiguity, among others.^2^, ^3^ The imaging appearance of the normal reproductive tract changes over the female's life, largely as a result of hormonal influence.^4^ The neonatal uterus is prominent, and the cervix is larger than the fundus (fundal–cervical ratio 1:2); in contrast, the prepubertal uterus generally has a tubular shape, and the pubertal uterus has the adult pear configuration, with the fundus larger than the cervix (fundal–cervical ratio 2:1).^2^, ^5^

In order to perform an accurate pelvic assessment, the examiner must be well trained and possess good knowledge of the appearance and size of the ovaries and uterus at different pediatric ages. Nevertheless, the availability of quantitative ranges helps to objectively define normal parameters. Several studies during the 1980s and 90s assessed the development of the ovaries and uterus throughout the childhood of healthy girls to provide reference values.^6^, ^7^, ^8^, ^9^, ^10^ However, there are some discrepancies between these early studies, possibly due to limitations in statistical analysis, the small size of the study populations or the formulas used for estimations.^11^ New formulas to estimate ovarian and uterine values in relation to other variables such as age were proposed,^12^, ^13^, ^14^ but up‐to‐date information is scarce, and genetic, social, and environmental characteristics may trigger variations with time in anatomic features. Puberty presently occurs at a younger age,^15^ and US technology has seen great improvements in recent years. In premature thelarche, a combination of clinical signs and uterine and ovarian measurements are routinely used to differentiate between this normal variant of puberty and central precocious puberty (CPP). For this reason, establishing a new updated reference range for PUS values in healthy girls is paramount to guarantee accurate diagnosis.

The primary objective of this study was therefore to determine the reference pelvic ultrasonography criteria (ovarian and uterine) in a population of healthy girls between 6 and 12 years of age. The secondary objectives were i) to correlate the PUS findings with pubertal status and bone age, and ii) to obtain tables with up‐to‐date uterine and ovarian measurements.

## 2 MATERIALS AND METHODS

### 2.1 Design and participants

This was a descriptive cross‐sectional study conducted in 221 healthy Caucasian girls between 6 and 12 years of age recruited from three schools selected by stratified random sampling in the region of Vallès Occidental (Spain) between 2017 and 2019. A pediatrician visited each school to provide written information about the study to parents/guardians and request the participation of girls aged 6 to 12 years who did not meet any of the exclusion criteria (genetic or endocrine disorders, metabolic disorders, history of abdominal or pelvic surgery, or any malformation). Parents/legal guardians interested in the study provided an email address to arrange an appointment. During this visit, the informed consent was signed; chronological age and anthropometric data of the participant (weight, height, and body mass index [BMI]) were collected; and pubertal Tanner stage was determined; PUS was performed to obtain ovarian and uterine measurements of the participant. If the parent/guardian agreed, an X‐ray of the wrist was carried out by two experienced pediatric radiologists to determine bone age according to the Greulich and Pile atlas method. Each family was subsequently informed of the participant's results; if any abnormality was detected, another visit was arranged. Data confidentiality was maintained at all times, and this study followed the procedures of the Declaration of Helsinki. The Local Ethics Committee of Parc Tauli Foundation approved this protocol (code 2017543). All participants gave consent to participate and for publication, and these statements are available.

### 2.2 Variables and procedures

**FIGURE 1:**
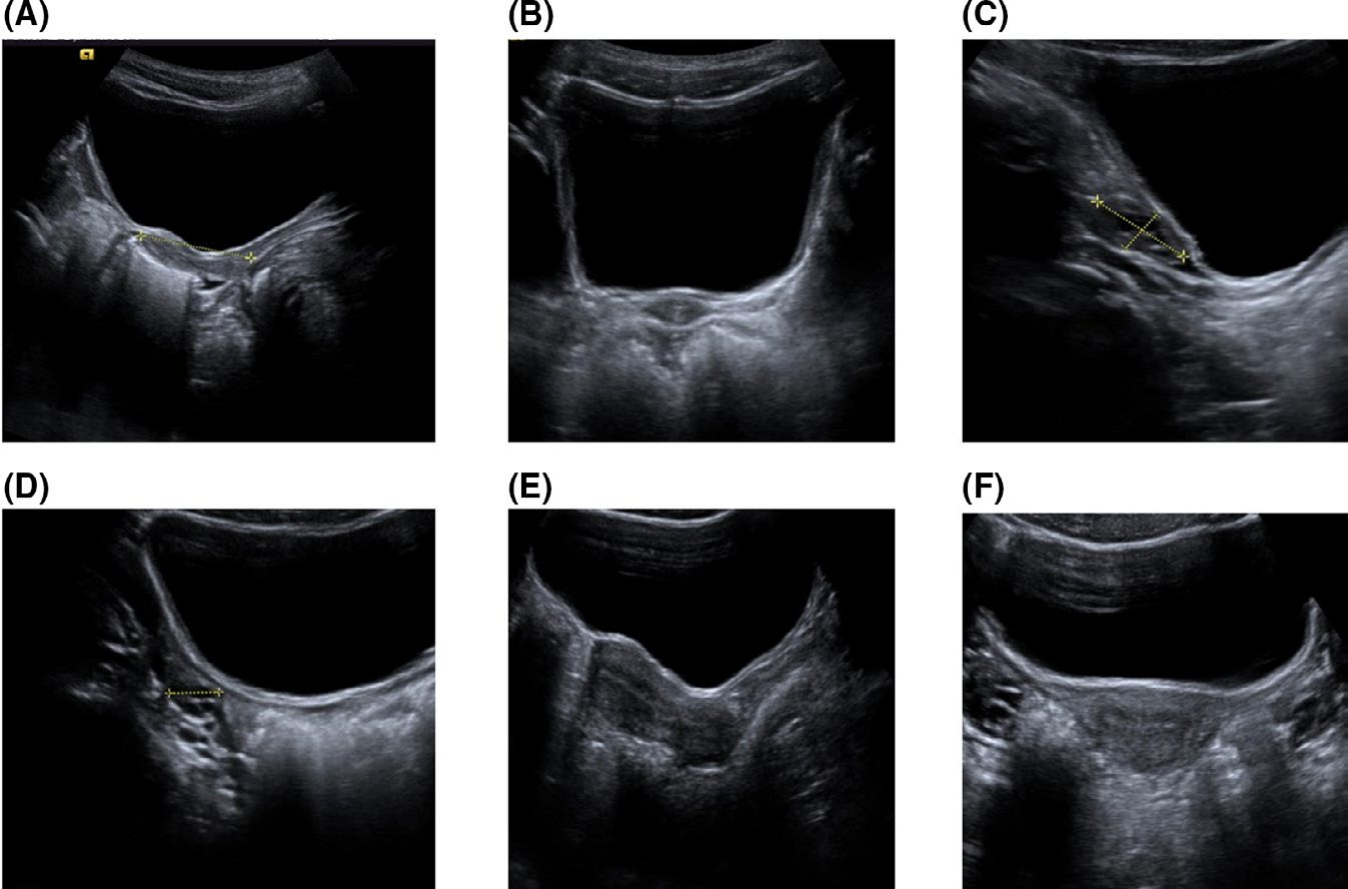
Selected images from a PUS examination in a 7‐year‐old girl that show measurements of: a) longitudinal and anteroposterior dimensions of the uterus, b) transverse dimension of the uterus, c) longitudinal and anteroposterior dimensions of the right ovary, and d) transverse dimension of the right ovary. Images e) and f) show a sagittal and an axial view, respectively, of a postmenarcheal uterus from a different examination in another girl

The variables included in the study were chronological age, bone age, weight, height, BMI, Tanner stage,^16^ ovarian transverse diameter, ovarian anteroposterior diameter, ovarian longitudinal diameter, ovarian volume, uterine length, anteroposterior diameter of the fundus, anteroposterior diameter of the cervix, and fundal–cervical ratio (FCR). Pubertal development was assessed according to Tanner staging by direct physical examination. This was performed by a pediatric endocrinologist with more than 20 years of experience in clinical practice. Transabdominal scans/PUS were obtained by two experienced pediatric radiologists and a pediatric radiology technician, using an ACUSON S2000 ultrasound system (Siemens Medical Solutions, Germany) with a 6‐MHz convex transducer. Subjects were given clear fluids to drink to fill the bladder and provide an acoustic window through which to examine the pelvic organs. Axial and sagittal views of the uterus and ovaries enabled measurement of uterine length and the anteroposterior diameter of the cervix and fundus (Figure 1). The characterization of the FCR was performed by a morphological analysis of the ultrasound images. In a sagittal view, a tubular shape of the uterus corresponded to a 1:1 ratio, whereas the diameter of the fundus being twice the diameter of the cervix implied a 2:1 ratio. The presence or absence of a midline endometrial echo was noted, and measurements of the longitudinal, anteroposterior, and transverse diameter of each ovary were obtained. Ovarian volumes were subsequently calculated using the prolate ellipsoid formula (Ovarian volume = length x width x height x 0.523). Interobserver agreement was checked using the variable ovarian volume in duplicate (left and right ovaries), and both observers noted similar results for the overall sample, with consistent results for each age‐group.

### 2.3 Statistical analysis

To calculate the sample size, we aimed to obtain a precision of *SD* *(Standard Deviation) =0*.25; 30 subjects per age were therefore required (210 in total). The quantitative variables were described using the mean, median, and *SD*. The Student's *t‐*test for paired data was performed to verify the absence of significant differences between left and right ovarian measurements; means were then calculated for further analyses. Bivariate analysis of ovarian and uterine measurements (response variables) according to chronological age, bone age, and Tanner stage (explanatory variables) was then conducted. When the explanatory variable was statistically significant, pairwise comparisons of group means were carried out using Tukey's range test. The estimation of bone age, ovarian volume, and uterine length to predict Tanner stage II (corresponding to thelarche) was assessed by means of receiver operating characteristic (ROC) curve analysis. A significance level of 5% was set for all tests, and all data were analyzed with SAS version 9.4, SAS Institute Inc., Cary, NC, USA.

## 3 RESULTS

The characteristics of the study subjects according to the Tanner stage are shown in Table 1. The girls classified in this study as Tanner stage II, which corresponds to thelarche, had a mean age of 9.7 years (*SD = *1.1) and mean BMI of 19.2 kg/m^2^ (*SD = *3.1) (Table 1).

**TABLE 1:** Characteristics of the study subjects according to the Tanner stage. Data are expressed as means ± standard deviations (*SD*) and medians (IQR).

	Tanner I N = 108	Tanner II N = 55	Tanner III N = 44	Tanner IV N = 13
Chronological age (years)				
Mean ± *SD*	7.5 ± 1.4	9.7 ± 1.1	11.1 ± 1.0	11.7 ± 0.5
Median (IQR)	7 (6–8)	10.0 (9.0–11.0)	11.0 (11–12.0)	12.0 (11.0–12.0)
Bone age (years)				
Mean ± *SD*	7.9 ± 1.4	10.1 ± 1.1	11.8 ± 1.2	13.8 ± 1.3
Median (IQR)	7.7 (6.8–8.9)	10.2 (9.1–11)	11.5 (11.0–13.0)	14.0 (13.0–15.0)
Weight (Kg)				
Mean ± *SD*	29.1 ± 7.2	38.6 ± 8.7	46.3 ± 9.9	57.6 ± 14.4
Median (IQR)	27.8 (23.5–34.0)	38 (31.0–45.0)	43.8 (37.9–53.0)	55.0 (46.5–64.8)
Height (cm)				
Mean ± *SD*	127.7 ± 8.9	141.3 ± 8.0	150.4 ± 7.0	160.4 ± 7.1
Median (IQR)	127.5 (121.2–134.4)	141.0 (137.1–147.9)	150 (144.8–155.9)	159.8 (156.8–164.7)
BMI (Kg/m^2^)				
Mean ± *SD*	17.6 ± 2.7	19.2 ± 3.1	20.4 ± 3.6	22.2 ± 4.4
Median (IQR)	16.9 (15.8–19.1)	18.9 (16.7–21.2)	19.3 (18.0–22.8)	21.1 (19.3–25.6)

Abbreviations: BMI, body mass index; IQR, interquartile range.

### 3.1 Pelvic organ measurements according to age and Tanner stage

**FIGURE 2:**
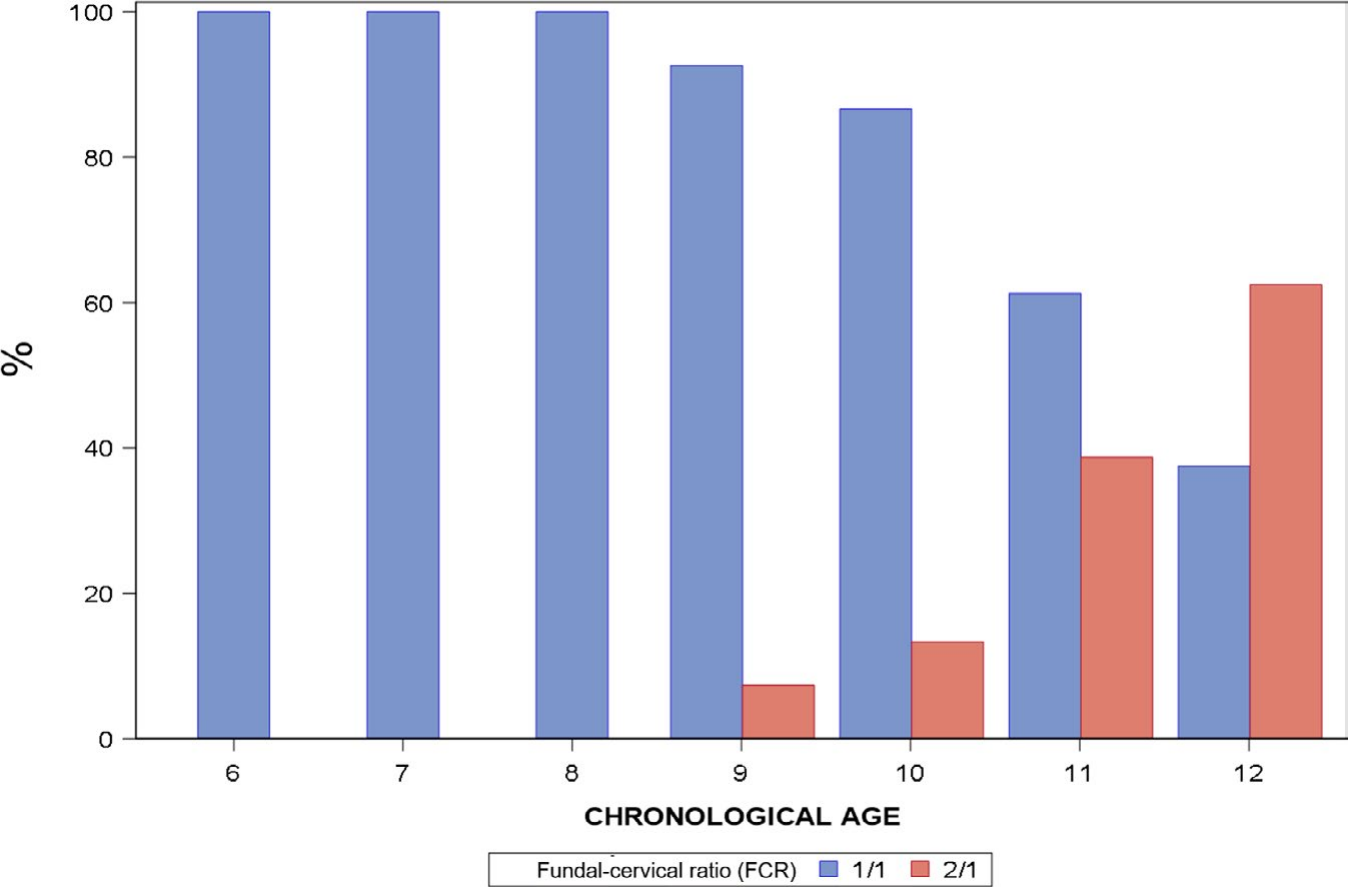
Distribution of the variable fundal–cervical ratio (FCR) according to chronological age in all study subjects

The study allowed reference values to be obtained for healthy girls aged 6 to 12 years for all PUS uterine and ovarian measurements assessed. The uterus and endometrial ultrasound images were seen in all patients, and both ovaries were identified in 95% of the girls, while only one ovary was identified in eight girls. Left and right ovaries presented similar results in all measurements, and subsequent analyses were carried out with the mean values. Mean PUS measurements according to chronological age are shown in Table 2. Progressive increases were observed in all the variables analyzed, so significant differences were found only when comparing the youngest girls with groups aged over 10. A significant change in uterine length (*p  *=  0.0020), ovarian transverse diameter (*p  *< 0.0001), ovarian anteroposterior diameter (*p  *=  0.0017), and ovarian volume (*p   *= 0.0014) takes place at 10 years of age in girls with normal development, whereas significant changes in the anteroposterior diameter of the fundus (*p *< 0.0001) and the cervix (*p *< 0.0001) occur at 11 years old (chronological age). After evaluating the distribution of the FCR in the different groups, we observed a change from 1:1 FCR to 2:1 FCR at 12 years of chronological age, when 62.5% of subjects presented a 2:1 ratio (*p * < 0.0001). Before that age, most subjects had a 1:1 FCR (Figure 2). The resulting bone age was the same as the chronological age to indicate when all parameters significantly change, except for the FCR, which presented the inflection point at 13 years of bone age (2:1 ratio in 85.71% of individuals [*p * < 0.0001]).

**TABLE 2:** Mean and median PUS measurements according to chronological age. Standard deviation (*SD*) for each mean value and minimum/maximum for each median are shown in parentheses.

Chronological age	6	7	8	9	10	11	12
N	25	30	23	28	30	31	24
Uterine length (cm)							
Mean ± *SD*	3.282 (0.363)	3.527 (0.373)	3.614 (0.453)	3.849 (0.510)	4.113 (0.687)	5.336 (1.321)	6.288 (1.050)
Median (min, max)	3.320 (2.265, 3.880)	3.540 (2.940, 4.212)	3.590 (2.600, 4.630)	3.885 (3.070, 4.950)	3.975 (2.700, 5.936)	5.226 (3.310, 8.178)	6.460 (4.600, 8.200)
Anteroposterior diameter of the fundus (cm)							
Mean ± *SD*	0.594 (0.159)	0.625 (0.145)	0.539 (0.123)	0.766 (0.328)	0.918 (0.462)	1.716 (0.895)	2.250 (0.624)
Median (min, max)	0.600 (0.300, 0.900)	0.600 (0.300, 0.900)	0.500 (0.300, 0.800)	0.700 (0.200, 1.600)	0.700 (0.100, 2.100)	1.440 (0.600, 3.600)	2.200 (1.100, 3.400)
Anteroposterior diameter of the cervix (cm)							
Mean ± *SD*	0.558 (0.140)	0.569 (0.119)	0.509 (0.104)	0.655 (0.246)	0.740 (0.187)	1.146 (0.360)	1.391 (0.309)
Median (min, max)	0.510 (0.300, 0.800)	0.600 (0.300, 0.800)	0.500 (0.300, 0.700)	0.600 (0.200, 1.300)	0.700 (0.500, 1.300)	1.200 (0.500, 1.900)	1.300 (0.900, 2.000)
Mean ovarian transverse diameter (cm)							
Mean ± *SD*	1.086 (0.182)	1.224 (0.203)	1.168 (0.248)	1.289 (0.207)	1.418 (0.288)	1.596 (0.237)	1.714 (0.311)
Median (min, max)	1.063 (0.841, 0.845)	1.175 (0.845, 1.691)	1.130 (0.882, 1.760)	1.277 (0.870, 1.646)	1.352 (0.985, 2.335)	1.585 (1.170, 2.337)	1.641 (1.312, 2.510)
Mean ovarian anteroposterior diameter (cm)							
Mean ± *SD*	0.925 (0.165)	0.979 (0.177)	0.951 (0.142)	1.077 (0.170)	1.190 (0.302)	1.434 (0.242)	1.555 (0.393)
Median (min, max)	0.920 (0.582, 1.278)	0.970 (0.691, 1.366)	0.920 (0.704, 1.310)	1.075 (0.790, 4.564)	1.099 (0.872, 2.484)	1.434 (0.923, 1.903)	1.512 (0.976, 2.535)
Mean ovarian longitudinal diameter (cm)							
Mean ± *SD*	2.129 (0.307)	2.361 (0.364)	2.421 (0.417)	2.700 (0.433)	2.742 (0.408)	3.162 (0.448)	3.359 (0.442)
Median (min, max)	2.119 (1.661, 2.940)	2.345 (1.734, 3.600)	2.370 (1.660, 3.135)	2.653 (2.026, 4.141)	2.620 (2.031, 4.008)	3.098 (2.499, 3.991)	3.349 (2.647, 4.050)
Mean ovarian volume (cm^3^)							
Mean ± *SD*	1.149 (0.410)	1.516 (0.550)	1.442 (0.573)	1.985 (0.670)	2.601 (2.007)	3.895 (1.396)	4.836 (2.116)
Median (min, max)	1.132 (0.474, 1.970)	1.345 (0.525, 2.759)	1.333 (0.689, 2.888)	1.907 (1.102, 3.563)	2.154 (1.146, 12.144)	3.506 (1.485, 8.229)	4.799 (2.279, 10.544)

Regarding PUS variables according to Tanner stage (Table 3), mean uterine length for Tanner stage II (corresponding to thelarche) was 4.065 cm (*SD  = *0.736), while mean ovarian volume was 2.466 cm^3^ (*SD  = *1.719). Additionally, 90.7% (*n* = 49) of girls in Tanner stage II had a 1:1 FCR.

**TABLE 3:** Mean and median PUS measurements according to Tanner stage. Standard deviation (*SD*) for each mean value and minimum/maximum for each median are shown in parentheses.

Breast development	Tanner I N = 108	Tanner II N = 55	Tanner III N = 44	Tanner IV N = 13
Uterine length (cm)				
Mean ± *SD*	3.475 (0.416)	4.065 (0.736)	5.594 (1.037)	6.934 (0.931)
Median (min, max)	3.940 (2.265, 4.590)	3.940 (2.600, 6.200)	5.380 (4.070, 7.600)	6.982 (4.891, 8.200)
Anteroposterior diameter of the Fundus (cm)				
Mean ± *SD*	0.610 (0.166)	0.865 (0.750)	1.866 (0.746)	2.683 (0.522)
Median (min, max)	0.600 (0.300, 1.200)	0.750 (0.200, 2.000)	1.600 (0.700, 3.600)	2.800 (1.800, 3.600)
Anteroposterior diameter of the cervix (cm)				
Mean ± *SD*	0.572 (0.135)	0.717 (0.257)	1.214 (0.342)	1.500 (0.214)
Median (min, max)	0.600 (0.300, 1.000)	0.700 (0.200, 1.300)	1.200 (0.600, 2.000)	1.500 (1.200, 1.900)
Mean ovarian transverse diameter (cm)				
Mean ± *SD*	1.189 (0.228)	1.367 (0.297)	1.621 (0.275)	1.781 (0.267)
Median (min, max)	1.159 (0.768, 1.900)	1.387 (0.840, 2.297)	1.585 (1.235, 2.510)	1.758 (1.402, 2.337)
Mean ovarian anteroposterior diameter (cm)				
Mean ± *SD*	0.984 (0.191)	1.167 (0.303)	1.422 (0.297)	1.677 (0.361)
Median (min, max)	0.969 (0.582, 1.915)	1.135 (0.695, 2.484)	1.351 (0.976, 2.105)	1.629 (1.021, 2.535)
Mean Ovarian longitudinal diameter (cm)				
Mean ± *SD*	2.369 (0.399)	2.700 (0.460)	3.195 (0.465)	3.530 (0.353)
Median (min, max)	2.350 (1.661, 3.600)	2.661 (1.660, 4.008)	3.228 (2.499, 4.141)	3.582 (2.864, 3.955)
Mean ovarian volume (cm3)				
Mean ± *SD*	1.466 (0.534)	2.466 (1.719)	3.989 (1.714)	5.550 (1.681)
Median (min, max)	1.385 (0.484, 2.976)	2.284 (0.672, 11.954)	3.474 (1.714, 10.544)	5.258 (2.482, 8.515)

Mean PUS measurements according to bone age are presented in the Supplementary material (Table S1) .

### 3.2 Predictive measures of thelarche (Tanner II)

The usefulness of ovarian volume, uterine length, and bone age to predict Tanner II was analyzed. All data obtained for sensitivity, specificity, positive predictive value (PPV), and negative predictive value (NPV) were above 75% for the three measurements (Table 4). The highest values were achieved with bone age and ovarian volume. Therefore, bone age, ovarian volume, and uterine length are good predictors to correctly categorize Tanner stage II, especially bone age and ovarian volume.

**TABLE 4:** Sensitivity, specificity, positive predictive value (PPV), and negative predictive value (NPV) for some measurements as predictors of Tanner stage II. Each value is shown as percentage with its 95% confidence interval in parentheses.

	Bone age	Ovarian volume	Uterine length
Sensitivity (%)	86.61 (80.30–92.92)	78.30 (70.45–86.15)	80.91 (73.57–88.25)
Specificity (%)	83.33 (76.30–90.36)	84.47 (77.48–91.46)	79.44 (71.78–87.10)
PPV (%)	84.35 (77.71–90.99)	83.84 (76.59–91.09)	80.18 (72.76–87.60)
NPV (%)	85.71 (79.02–82.40)	79.91 (72.42–87.40)	77.36 (69.39–85.33)

## 4 DISCUSSION

PUS is the preferred imaging modality for initial assessment of the morphology and size of the uterus and ovaries, although there are some discrepancies across different published studies. The different values reported in the literature for sonographic variables may have resulted from interobserver variation, the resolution of the sonographic equipment, and the degree of bladder fullness. Additionally, differences in the sample size, population, and methods of volume calculation in the individuals studied might have had an effect.^17^ As we mentioned, one important fact is the change in the morphology of the uterus over time. Although many authors have been using an ellipsoid formula, and most conclude that they have good correlation, it may be a mistake to use a simple equation to describe the volume over time. For this reason, some investigators have suggested that more attention should be paid to uterine length than to uterine volume, as uterine length correlates better with age.^5^, ^12^

This study evaluated ovarian and uterine measurements of healthy girls aged 6 to 12 from the region of Vallès Occidental (Spain) and how they correlate with chronological age, bone age, and Tanner stage. Characteristics such as chronological age, bone age, weight, height, BMI, and Tanner stage were analyzed for all study subjects. In cases of premature thelarche, having the reference characteristics for the healthy population together with the PUS results might help to categorize thelarche as normal or pathological. In fact, it has been suggested that the increase in weight and BMI could stimulate rapidly progressive puberty in cases with premature thelarche.^18^, ^19^ Our study provides up‐to‐date reference values in an autochthonous Spanish population for relevant measurements in clinical practice. This means progress for clinical practice, as most available datasets have been reported in the United States, where features such as BMI or mean age at the onset of puberty differ from those in our population. Moreover, environmental and genetic factors might have contributed to modify normal values for healthy girls in the last decades^15^; thus, these datasets could be obsolete.

As in previous studies, our study demonstrates that uterine and ovarian parameters grow with increasing age. Our data further showed that the increments in uterine parameters such as uterine length, anteroposterior dimensions of fundus and cervix, and ovarian volumes were significantly different between 10‐ and 11‐year‐old girls. This differs slightly from previous reports, where the significant change occurs at 9 or 10 years old,^11^, ^20^, ^21^ and could be in response to genetic, environmental, or timeline changes.

Another representative parameter supporting the PUS evaluation seems to be the FCR. In our study, most girls showed a 1:1 ratio until age 12, when it changes to 2:1. Several authors have measured this ratio to investigate whether there is a correlation between uterine morphology and thelarche, stating that it can be used to differentiate between prepubertal and pubertal status.^22^ Badouraki et al. showed how FCR was significantly greater in girls with central precocious puberty compared with healthy controls in the age‐group 8 – 10 years.^3^

Most available data regarding uterine and ovarian reference measurements can be considered obsolete over time, taking into account that the age of thelarche has fallen by 0.24 years per decade from 1977 to 2013.^15^ One of the strengths and unique features of this study was not including the uterine volume, as it does not seem accurate to use it as a reference value, given that the uterus is not a perfect sphere and other uterine measurements should be used instead. An advantage of this study was to prospectively recruit participants who did not necessarily have an indication for a US scan, therefore avoiding selection bias. Additionally, an added value of our study lies in providing clinical data of subjects such as BMI, Tanner stage, or bone age. Regarding the differences observed between studies, since US is an operator‐dependent technique, the protocol is crucial to obtain reproducible measurements.

We also observed a greater ovarian volume on the right side, although the difference was not sufficiently large as to consider the ovaries separately. While some authors have found significant differences,^5^, ^23^ many others have not.^11^, ^12^, ^13^, ^17^, ^24^ We hypothesize that this could be due to more difficult visualization of the left ovary, as it can be obscured by gas in the sigmoid colon. Finally, our results showed that ovarian volume, bone age, and uterine length are good predictors of Tanner stage II, corresponding with thelarche. Mean chronological and bone age of Tanner stage II girls was similar, and the predictive value of bone age for Tanner stage II was considerably high. Hence, chronological age is itself a good indicator to discriminate between normal or abnormal findings in a patient presenting Tanner stage II.

The main limitation in this study is the fact that US, despite being the most convenient technique for the study of pelvic organs, especially in the pediatric population, has its own limitations insofar as it is difficult to obtain reproducible and accurate measurements. In contrast, the approach of two highly experienced operators from a single center presents high homogeneity of measurements. Additionally, this constitutes the first European series of contemporary values for a restricted population, which is crucial to guarantee accurate diagnosis.

## 5 CONCLUSION

This study provides updated pelvic ultrasound reference values for uterine length, anteroposterior diameter of the fundus, anteroposterior diameter of the cervix, fundal–cervical ratio, ovarian transverse diameter, ovarian anteroposterior diameter, ovarian longitudinal diameter, and ovarian volume in a Spanish population of healthy girls aged 6 to 12. These reference values can be used in the clinical setting to improve the accuracy of differential diagnoses when facing presentations such as premature thelarche. Our results showed that bone age and ovarian volume in particular are good predictors of Tanner stage II. This is the first study to yield reference uterine and ovarian PUS values in Spain.

## CONFLICTS OF INTEREST

The authors declare no conflicts of interest relevant to this article.

## AUTHORS CONTRIBUTION

RC designed the project, performed the study, and participated in the manuscript; VB and CD are the radiologists; and MV and MP carried out fieldwork.

## Supporting information

Table S1

## Data Availability

Data are available on request due to privacy/ethical restrictions.
