# Supplementary material for: Updated reference values in pelvic ultrasonography for a Spanish population of healthy girls between 6 and 12 years old
Source: Endocrinol Diabetes Metab. 2021 Feb 4;4(3):e00233. doi: 10.1002/edm2.233 (PMC8279629; doi:10.1002/edm2.233)
Supplement: Supplementary file 1 — Table S1 [file EDM2-4-e00233-s001.docx]

**Table S1.** Mean and median PUS measurements according to bone age. Standard deviation for each mean value and minimum/maximum for each median is shown in parentheses.

| **Bone age** | | **5y + 9m** | **6y + 10m** | **7y + 10m** | **8y + 10 m** | **10y** | **11y** | **12y** | **13y** |
| --- | --- | --- | --- | --- | --- | --- | --- | --- | --- |
| **N** | | 14 | 28 | 38 | 25 | 44 | 34 | 12 | 25 |
| **Uterine length (cm)** | **Mean ± SD** | 3.311 (0.324) | 3.252 (0.331) | 3.482 (0.386) | 3.628 (0.391) | 3.953 (0.505) | 4.894 (0.862) | 5.805 (0.889) | 6.621  (0.974) |
|  | **Median**  **(min, max)** | 3.315 (2.603, 3.880) | 3.270 (2.265, 3.659) | 3.476 (2.600, 4.212) | 3.675 (2.600, 4.210) | 3.930 (2.980, 5.300) | 4.890 (3.391, 7.600) | 5.925 (4.600, 7.063) | 6.841  (4.100, 8.200) |
| **Anteroposterior diameter of the fundus (cm)** | **Mean ± SD** | 0.546 (0.119) | 0.631 (0.147) | 0.573 (0.159) | 0.591 (0.172) | 0.808 (0.273) | 1.362 (0.611) | 1.917 (0.639) | 2.554  (0.593) |
|  | **Median**  **(min, max)** | 0.560 (0.300, 0.700) | 0.650 (0.300, 0.900) | 0.600 (0.300, 1.000) | 0.600 (0.200, 0.900) | 0.700 (0.300, 1.600) | 1.300 (0.600, 3.600) | 1.700 (1.300, 3.400) | 2.7500  (1.100,  3.600) |
| **Anteroposterior diameter of the cervix (cm)** | **Mean ± SD** | 0.503 (0.114) | 0.602 (0.133) | 0.534 (0.114) | 0.558 (0.138) | 0.706 (0.185) | 0.977 (0.318) | 1.417 (0.266) | 1.433  (0.324) |
|  | **Median**  **(min, max)** | 0.500 (0.300, 0.700) | 0.600 (0.300, 0.800) | 0.500 (0.300, 0.700) | 0.600 (0.200, 0.800) | 0.700 (0.300, 1.100) | 1.000 (0.500, 1.800) | 1.300 (1.100, 2.000) | 1.400  (0.900,  2.200) |
| **Mean ovarian transverse diameter (cm)** | **Mean ± SD** | 1.073 (0.178) | 1.123 (0.218) | 1.185 (0.248) | 1.231 (0.220) | 1.362 (0.185) | 1.505 (0.292) | 1.683 (0.405) | 1.782  (0.377) |
|  | **Median**  **(min, max)** | 1.043 (0.846, 1.415) | 1.115 (0.816, 1.550) | 1.202 (0.840, 1.814) | 1.235 (0.888, 1.750) | 1.385 (1.010, 1.900) | 1.455 (0.768, 2.297) | 1.782 (1.200, 2.450) | 1.716  (1.228,  2.873) |
| **Mean ovarian vertical diameter (cm)** | **Mean ± SD** | 0.865 (0.168) | 1.006 (0.219) | 0.967 (0.196) | 0.970 (0.130) | 1.114 (0.156) | 1.361 (0.334) | 1.489 (0.342) | 1.621  (0.359) |
|  | **Median**  **(min, max)** | 0.925 (0.582, 1.091) | 0.985 (0.691, 1.915) | 0.957 (0.631, 1.366) | 0.970 (0.695, 1.193) | 1.133 (0.790, 1.387) | 1.368 (0.855, 2.484) | 1.331 (1.081, 2.105) | 1.631  (0.976,  2.535) |
| **Mean ovarian longitudinal diameter (cm)** | **Mean ± SD** | 2.085 (0.337) | 2.219 (0.321) | 2.333 (0.339) | 2.542 (0.369) | 2.651 (0.343) | 3.132 (0.480) | 3.200 (0.422) | 3.379  (0.406) |
|  | **Median**  **(min, max)** | 2.026 (1.739, 2.869) | 2.239 (1.661, 3.025) | 2.348 (1.660, 3.600) | 2.600 (1.748, 3.185) | 2.652 (1.720, 3.460) | 2.995 (2.499, 4.141) | 3.106 (2.673, 4.050) | 3.418  (2.647,  3.991) |
| **Mean ovarian volume (cm^3^)** | **Mean ± SD** | 1.048 (0.403) | 1.340 (0.535) | 1.435 (0.588) | 1.587 (0.425) | 2.092 (0.542) | 3.564 (1.984) | 4.434 (2.406) | 5.269  (2.175) |
|  | **Median**  **(min, max)** | 1.095 (0.484, 1.666) | 1.213 (0.516, 2.795) | 1.288 (0.620, 2.976) | 1.620 (0.811, 2.257) | 2.121 (1.087, 3.298) | 3.322 (1.009, 11.954) | 3.429 (2.306, 10.289) | 4.917  (2.130,  10.594) |
